# Dielectric Properties of Polyamides: Polyhexamethylene Adipamide and Polyhexamethylene Sebacamide

**DOI:** 10.6028/jres.065A.022

**Published:** 1961-06-01

**Authors:** Alexander J. Curtis

## Abstract

The dielectric relaxation in two polyamides has been studied over a temperature range from −100 to 175 °C and a frequency range from 50 c/s to 10 Mc/s. The effects of various thermal treatments on the relaxation behavior and density, X-ray diffraction, and intrinsic viscosity have been studied. The polyamides were poly(hexamethylene adipamide) and poly(hexamethylene sebacamide). Four relaxation phenomena have been identified. Mechanical relaxation processes are compared with the dielectric phenomena and possible molecular mechanisms are discussed.

## 1. Introduction

The dielectric properties of polyamides have received widespread interest in recent years. Boyd [1]^1^ has reported measurements of the dielectric loss of poly(hexamethylene adipamide), 66 nylon, and discussed in detail the high temperature relaxation process in terms of molecular structure. McCall and Anderson have described measurements on a series of seven linear polyamides [2], and Rushton and Russell [13] have studied 66 nylon. The much earlier measurements of the dielectric properties of a number of polyamides by Baker and Yager [3] should also be mentioned.

The polyamides, or as they are commonly called, the nylons, have attracted the attention of a wide group of investigators for a number of reasons. Quite aside from their commercial importance, the nylons are of interest because they possess a highly complex structure and, as expected, have a complicated dielectric relaxation spectrum. The crystalline regions of 66 nylon and of 610 nylon, poly(hexamethylene sebacamide), have been studied by means of X-ray diffraction by Bunn and Garner [4]. It was shown that in the highly ordered crystalline regions, the polymer molecules lie in sheets in which adjacent molecules are joined through hydrogen bonds involving amide protons and carbonyl groups. The two principal lateral spacings determined from X-ray diffraction are, therefore, related to the interchain spacing within the hydrogen-bonded sheets and the intersheet spacing. It has been reported by a number of observers [5, 6, 7] that these two spacings merge and become indistinguishable above some temperature well below the melting point: in 66 nylon this temperature was reported to be 160 °C while the crystalline melting point was observed at 260 °C [6]. The structure above the transition temperature apparently corresponds to a disordered crystal in which the molecules have some rotational freedom about their long axes. It has also been shown that it is possible to obtain samples of nylon in which part or all of the ordered regions are in this rotationally disordered condition at room temperature [7]. This structural feature has been discussed in detail by Sandeman and Keller [8].

The present study of dielectric relaxation in two polyamides, 66 nylon and 610 nylon, is an attempt to correlate dielectric properties with pretreatment variables and in turn to show how these are related to such measurable quantities as crystallinity, moisture content, and molecular weight.

## 2. Experimental Details

### 2.1. Dielectric Measurements

**Figure 1:**
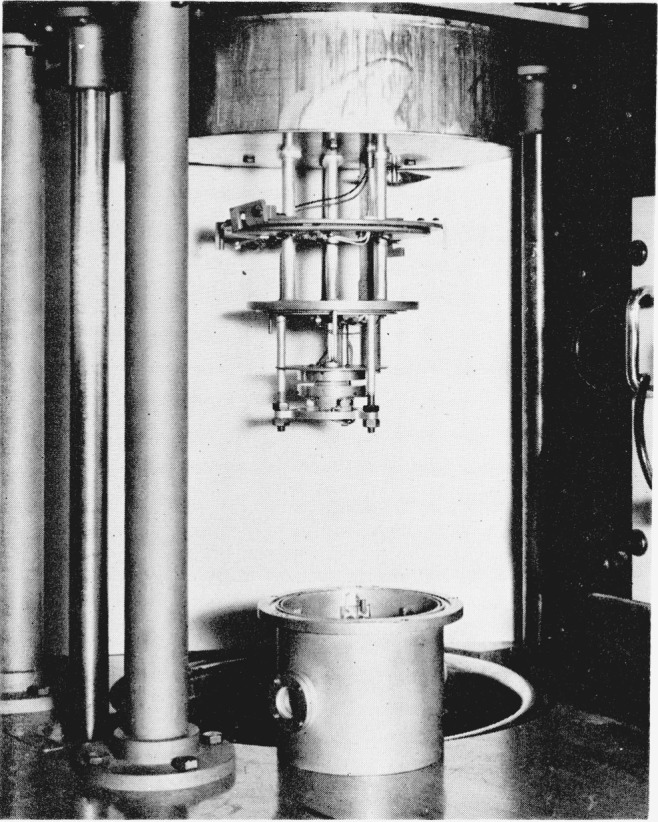
Cell for dielectric measurements shown with sample between electrodes.

All the dielectric measurements reported here were made with a cell specifically designed for measurements on disk-shaped solids, (fig. 1). All measurements were two-terminal. There is no reason to suspect that the present results, which are consistent with three-terminal measurements on similar materials [1], would have been different if three-terminal measurements had been made.

In figure 1 the cell is shown with the protective enclosure removed. In use, the electrodes were surrounded by this enclosure with heaters built into the walls to provide necessary heating. Temperature control was effected by independent sets of heaters in the top, the sides, and bottom of the enclosure. Thermocouples at the side and top were sensing elements for the controller. Two calibrated chromel-constantan junctions, one below the lower electrode and the other at the upper electrode, served to measure the temperature. The temperature difference between the upper and lower electrodes was never greater than a tenth of a degree at any of the temperatures at which dielectric measurements were carried out.

Below room temperature the entire cell and enclosure were surrounded by a large Dewar cylinder containing either dry ice or liquid nitrogen, without actually immersing the enclosure in the coolant. The controller supplied whatever current was necessary to maintain the desired temperature. Dry nitrogen was kept flowing through the cell at all times during measurements. At high temperatures, this prevented oxidation of the samples and at low temperatures it prevented condensation of moisture in the system.

Dielectric measurements from 50 c/s to 100 kc/s were carried out using a modified General Radio Schering Bridge (716–C). The higher frequency measurements up to 10 Mc/s were made using a Boonton Q-meter (Type 260–A). The details of the modifications and calibrations will be published elsewhere [9]. The precision and accuracy of the data reported here are in part a function of the dimensions of the samples used. Thus, although capacitance can easily be measured to better than 0.1 percent in almost all cases, the equivalent vacuum capacitance of a sample, as computed from the measured dimensions may involve greater uncertainties, which enter into the computation of each dielectric constant value. In order to reduce probable errors, samples having large equivalent vacuum capacitance were chosen for the low temperature measurements where dielectric constant and loss were relatively small and, correspondingly, samples of small equivalent vacuum capacitance were selected for the high temperature measurements, where extremely large values of dielectric constant and loss were encountered.

For samples having properly selected dimensions, it is estimated that the precision of the measurements on 66 nylon was 0.1 percent in the dielectric constant and 5 percent in the loss index. The measurements on 610 nylon involved the use of a more limited number of samples and similar precision was estimated for all but the low temperature measurements. The errors at low temperatures may have been as great as 1 percent in the dielectric constant, and as large as 10 percent in the loss index.

The principal factor involved in causing errors in measurement of dielectric properties of solids, aside from pretreatment and impurities, is the computation of the equivalent vacuum capacitance of a given disk. The error has been greatly reduced by measuring the diameter of each disk sample with a traveling microscope and computing the thickness from the diameter, the sample weight, and specific volume. This technique which has been described elsewhere [9] has been shown to give equivalent vacuum capacitances to better than 0.1 percent.

### 2.2. Preparation and Characterization of Specimens

The specimens of 66 nylon were pressed from a batch of nylon, in the form of chips, supplied by Dr. Albert Goodman and Miss Helen Anderson of the Du Pont Company. Disk shaped specimens were formed in a special mold designed for this purpose. In this mold, the nylon chips were melted under vacuum and pressed into disks. Because of the large heat capacity of the mold, it was not possible to cool the samples rapidly; generally it took about two hours to cool the mold and sample to room temperature. Because of this unavoidable annealing process, all the 66 nylon samples were quite highly crystalline, as indicated below.

The samples of 610 nylon were supplied by Dr. D. W. McCall of Bell Telephone Laboratories. These samples were from the same lot of polymer used in his work [2], and were supplied as injection molded disks. Some samples for the present study were formed by machining these disks to suitable dimensions. Others were formed by remelting and molding as in the case of the 66 samples.

All the samples were stored in a dessicator over phosphorus pentoxide. Before electrodes were applied, the samples were heated to about 110 °C in an evacuated container (*circa* 5–10×10^−3^ mm) for three days. Evaporated gold electrodes were applied to most of the samples. Painted silver electrodes were used in some cases. The latter type of electrodes has been found to introduce a very small extraneous dielectric loss in previous studies [23], but this effect is certainly negligible in measurements of a material as lossy as the polyamides. In the present study, samples with the two types of electrodes gave identical results. After the electrodes were applied, the samples were again heated to 110 °C in vacuum for about one day. A variety of further annealing processes were used for the various samples with consequent variations in physical properties, as indicated below.

**Figure 2:**
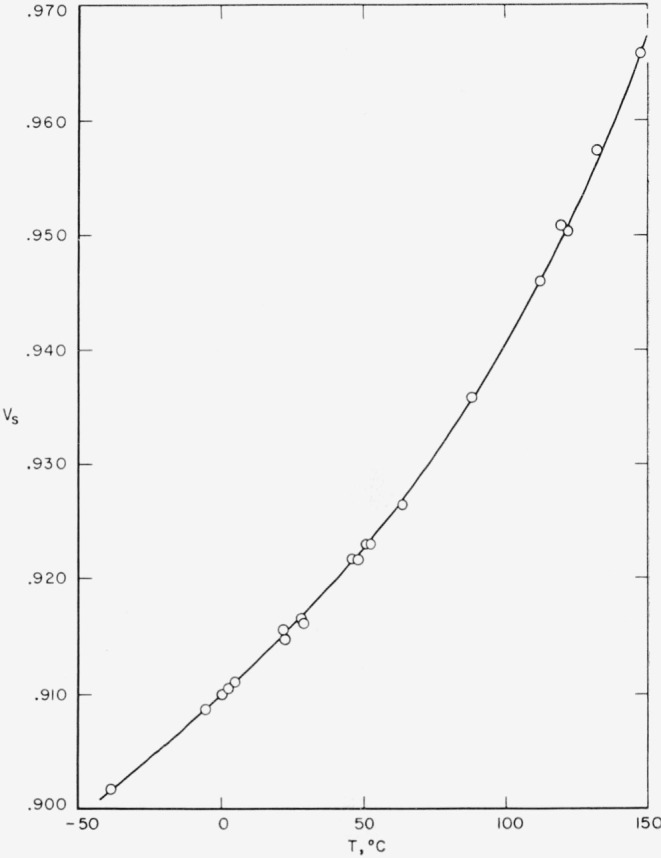
Specific volume (cm^3^/g) of 610 nylon as a function of temperature.

Specific volume measurements were carried out by a buoyancy technique. The measurements at room temperature and lower were carried out in *n*-heptane, while those at higher temperatures were in a silicone oil bath (Dow Corning “710”). Specific volumes were determined by comparing the measurements with those made simultaneously on a piece of fused silica. The apparatus employed was that described by Hoffman and Weeks [10]. Results obtained at room temperature (23 °C), together with specimen descriptions, are given in table 1. A typical plot of specific volume as a function of temperature used for the determination of thickness is shown in figure 2 for sample 610–8. Degrees of crystallinity were computed on the basis of the specific volume scale of Starkweather and Moynihan [11]. It is worth noting that very reproducible specific volumes were obtained for various specimens when nearly identical heat treatments were employed.

Intrinsic viscosities were obtained from measurements on *m*-cresol solutions at 30 °C. These measurements were undertaken to check on possible alterations in molecular weights resulting from the various treatments involved in the preparation of the specimens. The data are listed in table 2, together with sample descriptions. End group analysis gave a number average molecular weight of 16,000 for the 66 nylon chips as received. (Analysis showed 98 moles carboxyl end groups and 38 moles amine end groups per 10^6^ g of polymer.)

Flory [12] has developed the following relationship between number-average molecular weight (determined by end group analysis) and intrinsic viscosity in *m*-cresol for 66 nylon:

$${\bar{M}}_{n}=17,800[\eta ]-3,300.$$

The above expression demonstrates the well-known monotonic relationship between molecular weight and intrinsic viscosity for polymers. Using the intrinsic viscosity value obtained on polymer “as received” from table 2 (1.18), one obtains ${\bar{M}}_{n}=17,700$ which is in fair agreement with the value obtained from end group analysis.

It is evident from the data in table 2 that the various heat treatments carried out to dry the specimens have caused a definite increase in molecular weight. This effect will be dealt with subsequently.

**Figure 3:**
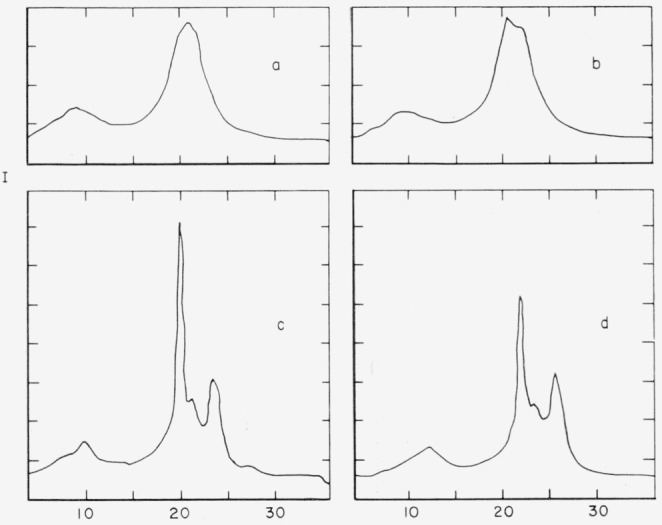
Relative X-ray intensity versus 2θ, (nickel-filtered copper K radiation). 6–10 Nylon; (A) injection molded, no heat treatment; (B) after five days in vacuum at 130 °C; (C) melted in vacuum, cooled slowly in mold; (D) injection molded, heated 1 day in vacuum to 210 °C.

The X-ray diffraction properties of the various samples were also measured with a North American Phillips diffractometer. Sample traces of X-ray intensity as a function of diffraction angle are shown in figure 3. These, and specific volume data, show that high temperature annealing is necessary to obtain maximum crystallinity in these polyamides.

**Figure 4:**
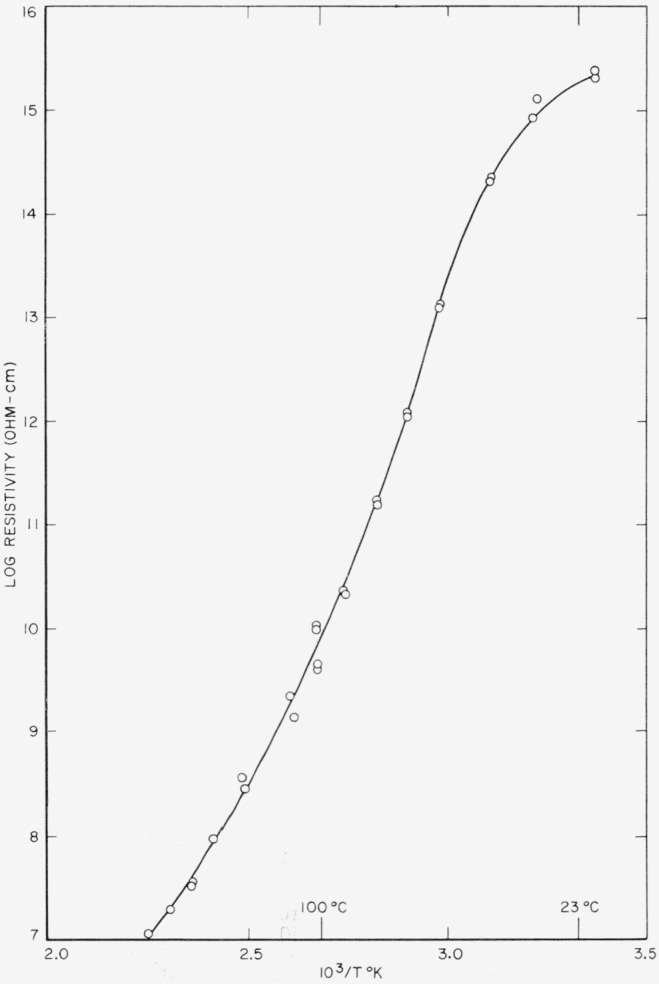
D–C resistivity of 66 nylon as a function of the reciprocal temperature.

The d-c resistance measurements were made with a Beckman Ultrohmeter with an external 200 v potential for resistances greater than 10^9^ ohms. Resistances below this value were measured with a General Radio Type 544–B megohm bridge. In the temperature region where the d-c conduction was of particular interest, i.e., above about 80 °C, it was found that there was such a large drift in resistance after applying the measuring potential that the results were of questionable value and low reproducibility. The data shown in figure 4 are computed from the only set of measurements made.

### 2.3. Experimental Results

The dielectric constants and losses of the various 66 nylon specimens are listed in table 3. Similar measurements on the 610 nylon samples are given in table 4. Thermal history of each specimen is briefly described in the tables.

There are several cases of nearly identical samples. The differences in the dielectric properties of these samples reflect the difficulty of obtaining semicrystalline polymer samples in identical physical states: for example, nylon specimen 610–1, after drying at 210 °C, had ϵ′ = 8.311 and ϵ″ = 0.950 while specimen 610–7, given similar treatment had ϵ′ = 8.391 and ϵ″ = 0.984, all measured at 1 kc/s and 80 °C.

The data in table 3 have not been corrected for the effect of d-c conduction on observed dielectric loss. As noted above, in the temperature region in which this correction would become significant, the measurement of d-c resistance becomes somewhat arbitrary. The significance of d-c conduction will be discussed below. In this particular case, we prefer not to alter the data in an arbitrary manner by using an assumed value of the d-c conduction.

## 3. Discussion of Results

**Figure 5:**
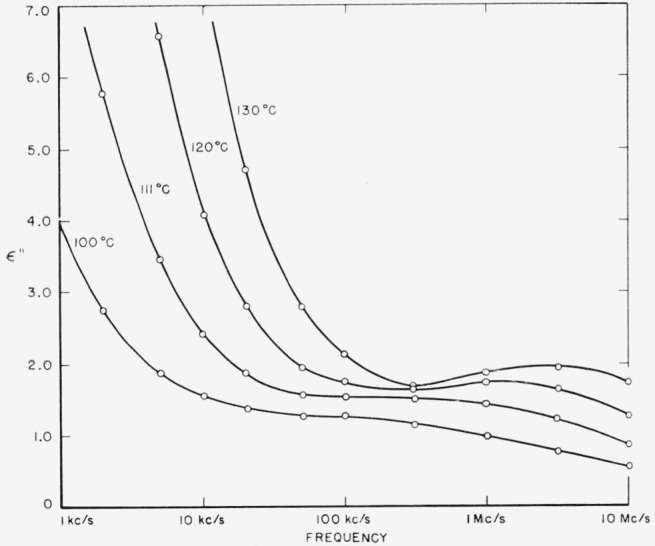
Dielectric loss index of 66 nylon as a function of frequency at high temperatures. The high-temperature dipole relaxation process appears as a maximum at the high frequency end.

**Figure 6:**
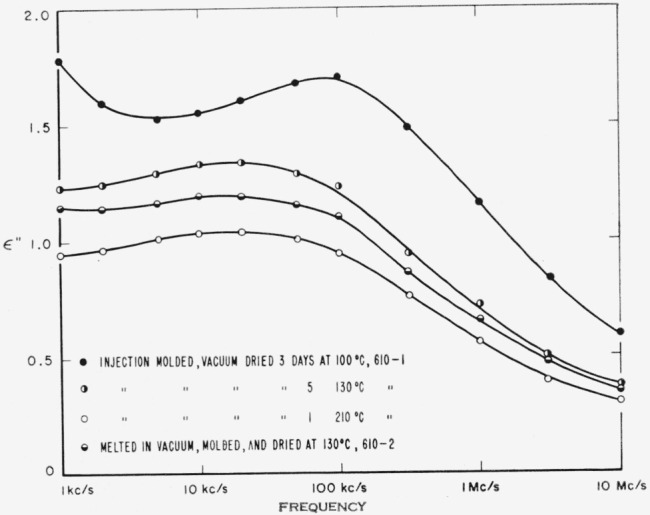
Dielectric loss of 610 nylon at 80 °C as a function of frequency.

**Figure 7:**
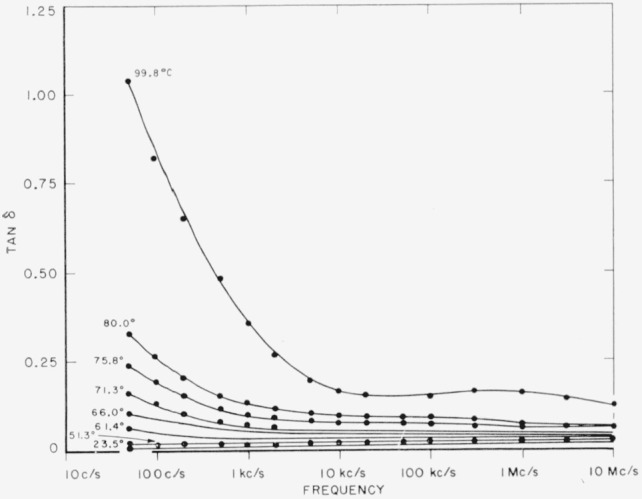
Loss tangent of 66 nylon as a function of frequency.

**Figure 8:**
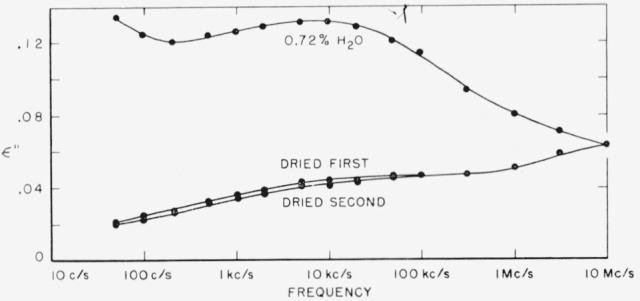
Dielectric loss index of 66 nylon at 23 °C as a function of frequency.

**Figure 9:**
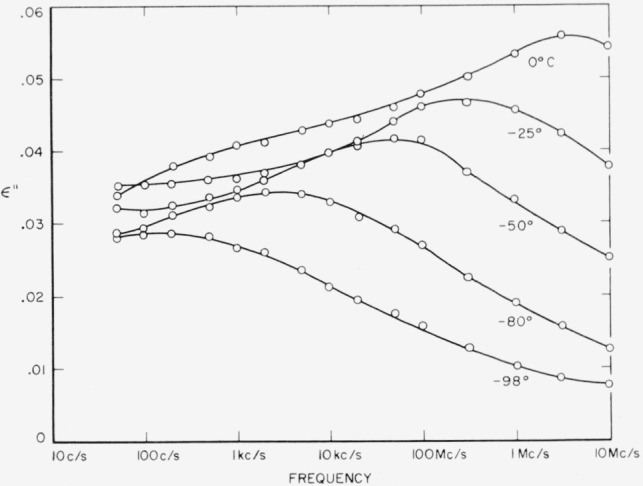
Dielectric loss index of 66 nylon at low temperatures as a function of frequency.

The important features of the dielectric relaxation behavior of the two polyamides examined in this study may be summarized as follows:

1. Both polymers exhibit a relaxation phenomenon which appears as a loss maximum at temperatures above about 80 °C and at frequencies above 1 kc/s. Some of the data from tables 3 and 4 are plotted in figures 5 and 6. It is seen that the relaxation process is similar in the two materials. This is the same relaxation effect identified as the “*α*” process by Boyd [1] and studied in detail by him in 66 nylon. It is also the same as that studied by McCall and Anderson [2] in 66 nylon as well as in six other polyamides.
2. Above about 50 °C, both polymers exhibit a low frequency polarization as manifested by a large dielectric constant and also a large loss. The appearance of this process is clearly shown by some of the data from table 3 for 66 nylon as plotted in figure 7. This phenomenon has been observed and reported previously [1, 2, 3], and is also apparent in figures 5 and 6.
3. There is a relaxation process observable at room temperature at about 10 kc/s in both systems. This process virtually disappeared in the case of 66 nylon after prolonged drying. The relaxation process could be made to reappear after absorption of very small quantities of water, and then to disappear again on drying as shown in figure 8. In the case of 610 nylon there was no case in which, even after prolonged drying, this relaxation process was completely removed. Rushton and Russell [13] have reported dielectric measurements that showed the pronounced effect that small amounts of water have on this process is 66 nylon, but in no case did they observe an absence of a maximum in their dielectric loss curves.
4. There is a dipole relaxation process observed between −100 °C and 0 °C in the frequency range studied here. This process, which has not been previously studied by dielectric methods, is present in both polymers, and is characterized by an energy of activation of about 13 kcal per mole of active dipole. Some of the low temperature data for 66 nylon which exhibit the loss peak due to this process are plotted in figure 9. This relaxation phenomenon is quite probably related to the mechanical relaxation observed in these and other polyamides by Woodward and coworkers [14], Willbourn [15], and Illers and Jenckel [16].

Although further investigation will be necessary before an unambiguous explanation in terms of molecular structure for all of these phenomena can be given, it is possible at this time to rule out some possibilities and to suggest others.

### 3.1. High Temperature Dipole Relaxation Process

The high temperature relaxation process seen in figures 5 and 6 has been extensively studied by Boyd [1] and McCall and Anderson [2], who referred to it as *α*′. The results presented here are in no way contradictory to their results. The absence of a clear maximum in the loss index at 80 °C, or even 100 °C, in 66 nylon indicates that the samples studied here were more highly ordered than those used by Boyd. He found a crystallinity of 45 percent while the samples used in this work were 57 percent. These values can be compared, since specific volumes and the same crystallinity scale were used in each case.

The relationship between order, thermal treatment, and the magnitude of the high temperature dipole relaxation process for 610 nylon may be seen by comparison of figure 6, which shows the loss indexes of several specimens of different degrees of order, and figure 3, which shows the X-ray diffraction intensities of the same specimens. The high temperature loss peak is evidently associated with disordered regions. The specific volume data for these specimens also supports this interpretation.

Sandeman and Keller [8] have pointed out the existence of a disordered crystalline phase in these polyamides. If such a structure exists as a separate phase in the polyamides, it would seem likely that a dipole relaxation phenomenon distinct from that for the even more disordered amorphous phase might be observed. The high temperature relaxation process could be associated with such a disordered crystalline phase. Boyd’s observation that cross linking by high energy electron irradiation caused a progressive disappearance of this dielectric relaxation process in 66 nylon, with very little effect on the relaxation time, can more readily be understood in terms of such a phase. It is reasonable to suppose that a few cross links in a disordered crystalline region would more effectively prevent chain rotation than in the amorphous phase. Chemical cross linking in amorphous polymers is known in some instances to raise the glass transition temperature [24]. If this relaxation process were related to such a transition, one might have expected a definite lowering of the frequency of the loss maximum at a given temperature due to cross linking. The data presented in this study do not prove or substantiate the hypothesis that the high temperature relaxation is due to a disordered crystalline region; they do indicate that this relaxation process is definitely not associated with the highly ordered regions of these polyamides, in agreement with both Boyd, and McCall and Anderson.

### 3.2. High Temperature, Low Frequency Polarization

The low frequency polarization process that is evident above about 50 °C in both 610 and 66 nylon (see, for example fig. 7 for 66 nylon) has a number of interesting features. The very high loss indexes observed at lower frequencies in both polyamides above this temperature cannot be entirely accounted for in terms of d-c conduction attributable to a simple ionic mechanism. This is readily shown by simple computation from the measured d-c resistance. For example, for 66 nylon at 100 °C, the contribution to the loss index due to d-c conduction is approximately 4.0 at 100 c/s, while the observed loss indexes for the various specimens listed in table 3 in this frequency range are between 15.29 and 22.08, depending on the amount of annealing and drying. This point is further demonstrated by the tremendous rise in dielectric constants. It is noteworthy that the highest loss index at 100 °C and 100 c/s is observed for specimen 66–6 before drying (22.08) while the lowest value is for 66–2 which had been dried (15.29). The value for specimen 66–6 closely approached this latter value after drying (15.95).

The high dielectric constants and loss indexes are evidently not simply due to electrode polarization; if this were the case, the values of dielectric constant and loss would be roughly proportional to sample thickness under comparable conditions.^2^ The present data, which do show considerable sensitivity to thermal history of the samples show no such trend. Compare for example, the high temperature data in table 3 for samples 66–1 and 66–2 in which the ratio of thickness of 66–1 to 66–2 is 0.926. At about 100 °C and 100 c/s for example, for 66–1 and 66–2, the dielectric constants were 20.56 and 18.60 respectively. In this case the comparison shows, as do the data at room temperature, that 66–2 was more nearly dry than 66–1. This low frequency polarization, then, must be largely a bulk property of these polyamides. Consideration of the dielectric properties of the two types of nonpolymeric substances mentioned below suggest the reasonableness of this conclusion.

Hoffman and Smyth [17] have described measurements of the dielectric properties of long chain, solid alcohols. A number of these compounds exhibit the interesting property of existing over some temperature range in “rotator” phases. In these rotator phases, the molecules exhibit hindered rotation with consequent dipole orientation. The hydroxyl groups in the crystal structure are arranged in sheets and are joined by hydrogen bonds. In the rotator phases, these hydrogen bonds occasionally break and reform to allow molecular orientation. The dielectric constants and losses were unusually high for these materials, and both quantities increased by as much as a factor of four in passing from the liquid to the rotator solid. These losses were interpreted in terms of a large proton drift mobility in the sheets of hydroxyls in the disordered rotator phase. The high dielectric constant was evidently a result of polarization due to a bound anion together with cation (proton) mobility.

Leader and Gormley [18, 19] have reported measurements of a number of amides and *N*-substituted amides in the liquid state. They observed exceedingly high dielectric constants for the *N*-monosubstituted amides: *N*-methylformamide had a dielectric constant of 182.4 at 25 °C while that of *N*-methylproprionamide was 172.2 at the same temperature. They observed that unsubstituted amides had lower dielectric constants, 110 for formamide and 74 for acetamide. The disubstituted amides had much lower dielectric constants: 36.71 for *N*, *N*-dimethylformamide and 37.78 for *N*, *N*-dimethylacetamide. It is clear that the polyamides under consideration have structures analogous to those of the monosubstituted amides. It might be expected, therefore, that in the liquid state, the amide groups of polyamides might associate into orientable structures of high dipole moment due to hydrogen bonding similar to those proposed by Leader and Gormley for the monosubstituted amides.

These hypotheses suggested a number of further experiments on the nature of the low frequency polarization mechanism, two of which will be briefly described here. It was thought that a slow relaxation process such as this might be “frozen in” at sufficiently low temperatures, resulting in a very slowly decaying polarization. In effect, it was thought that polyamides should make unusually strong electrets. The classical electret forming treatment was given to some samples: the sample disk was heated up to about 125 °C and a d-c electric field applied. Then, while maintaining the field, the sample was slowly cooled. Strong electrets were formed in this manner with both 66 and 610 nylon, and the electric field remained for many months when stored at room temperature. On reheating above about 80 °C, a small current could be withdrawn from the specimen over a period of hours, and resulted in the disappearance of the electret field.

It was only after these observations had been made that it was found that Wieder and Kaufman [20] had noted the strong electret forming properties of nylon some years previously.

It is also interesting to note that when a d-c field was applied at elevated temperatures to a specimen on which evaporated gold electrodes had been formed that, although the side towards the positive electrode appeared unchanged, the gold on the side toward the negative electrode was loose and readily flaked off. This phenomenon might well be due to the formation of hydrogen gas due to the electrolysis of the current carrying protons.

### 3.3. Water-Sensitive, Room Temperature Process

The relaxation process observed at about 10 kc/s at room temperature is clearly very sensitive to moisture. In figure 8, it is evident that, at least for 66 nylon, it is possible to remove enough of the water to cause the relaxation process to be almost completely absent. In the case of the 610 nylon, there was a maximum in the loss index curve for all specimens studied. However, careful drying greatly reduced the magnitude of the maximum in this case also.

It is noteworthy that although exposure of a dried specimen to moisture caused a reappearance of the maximum in 66 nylon, such exposure did not alter the appearance of the high temperature *α*′ process in these highly crystalline specimens. (In table 3 there was no loss maximum in sample 66–2 containing 0.69 percent water at 100 °C although the losses are generally higher than in comparable dry samples. This overall increase in loss is in agreement with the already noted effects of moisture on the low frequency, high temperature polarization process.) Thus, since the measurements of Rushton and Russell [13] and of Boyd [1] indicate a loss maximum at room temperature in their driest samples, it seems doubtful that all water had been removed.

The strong hydrogen bonding character of the amide groups in these polymers makes understandable their tenacious absorption of water. This effect is aggravated by the nature of the mechanism of forming these polymers, which results in formation of water as a by-product, and the fact that waterforming end groups (amine and carboxyl groups), remain after polymerization to a finite molecular weight. Thus, chemical equilibrium predicts the presence of small quantities of water which are inversely proportional to the molecular weight of the polyamide. The intrinsic viscosity results indicate that the most severe drying conditions have resulted in a shift of the equilibrium to higher molecular weight and, of course, the removal of some water. However, the fact that this dielectric relaxation process is not due to the end groups themselves but is due to water associated with the polymer, is shown by the fact that it may be made to reappear immediately on absorption of small amounts of water. Hydrolysis to reform end groups under the conditions used for these experiments is very improbable.

The fact that the relaxation process under consideration appeared in all the 610 nylon specimens apparently indicates that none of these were as dry as the 66 nylon. It is also significant that the highest intrinsic viscosity ([*η*] = 1.33) observed for any of the 610 nylon specimens was significantly lower than that for any of the 66 nylon specimens used in dielectric measurements ([*η*] = 1.69). Comparison of intrinsic viscosities of different polymers cannot be absolutely quantitative where other information is lacking, but in this case, where the chemical structures are so closely similar, it seems safe to say that the molecular weights of the 610 samples were lower in all instances than the 66 samples used for dielectric measurements.

If one makes certain simple assumptions, it is possible to compute the amount of dispersion in the dielectric constant to be expected from a given quantity of water. Assuming that the water absorbed in the nylon has the same polarizability as in the pure state, for which a dielectric constant of 78 is generally quoted at room temperature (21), then one might expect 0.72 percent of water to increase the low frequency dielectric constant by about 0.56. The data in table 3 for specimen 66–3 shows a difference in the dielectric constant of about 0.54 with and without this amount of water at 50 c/s. The difference in the dispersion of the dielectric constants in the frequency range studied, namely 50 c/s to 10 Mc/s, is 0.506 for the specimen with water and after drying. These figures demonstrate the reasonableness of the proposal that this relaxation process is due to the water-polymer complex. (Pure water shows dielectric dispersion in the microwave region at this temperature.)

**Figure 10:**
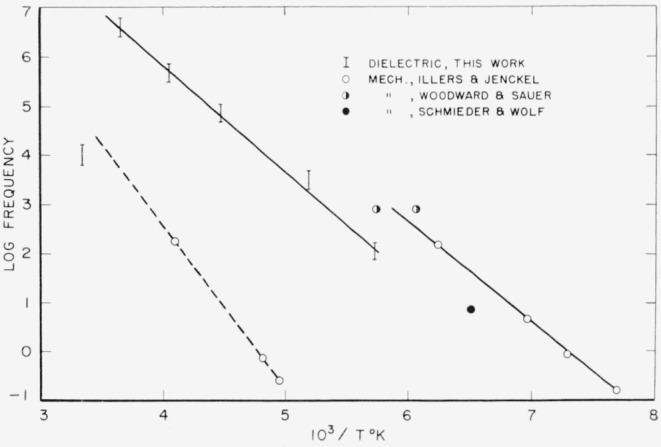
Frequency of loss maximum of 66 nylon as a function of reciprocal temperature. Water or β process indicated by dashed line, low temperature process by solid line.

This water-sensitive dispersion region is almost certainly closely related to the so called *β* process observed in mechanical measurements of polamides [14, 16], as noted earlier. Some of the mechanical data of Illers and Jenckel [16] are shown in figure 10, together with one dielectric point. In this plot of frequency of loss maximum against reciprocal of absolute temperature, it is evident that there is a correspondence between the mechanical *β* process and the room temperature dielectric loss maximum. It is evident from the dielectric and mechanical measurements that the relaxation process is characteristic of the water-polyamide complex and not of the pure polymer. The opinion has been expressed that a small quantity of swelling agent, such as water in this case, does not result in a new relaxation process but rather modifies processes characteristic of the pure polymer. In this case, such a view is not substantiated, and it is worth pointing out that Scheiber and Mead [22] have reported a similar situation with respect to the effect of water on dielectric dispersion in poly (methyl methacrylate).

### 3.4. Low Temperature Relaxation Process

The low temperature relaxation process observed in these data, as plotted in figure 9, is evidently related to the mechanical relaxation process observed in the same temperature region [14, 16]. The plot of the frequency of the maximum loss versus reciprocal of absolute temperature for the 66 nylon data is shown at the right-hand side of figure 10. The activation energy is 13 kcal/mole. Also included in this figure are some of the mechanical relaxation data of Illers and Jenckel, together with those of Woodward and coworkers, on what they have called the *γ* process. It is evident that the dielectric data do not exactly match the mechanical data, the latter having an activation energy of 9 kcal/mole. Further, the displacement of the two sets of data indicate a difference in entropy of activation. To understand these differences, it is first necessary to decide what molecular motions are responsible for this process.

The mechanical relaxation process has been widely attributed to motions of the hydrocarbon portions of the polymer chains. Various arguments have been proposed to support this hypothesis [15]. The present results indicating the existence of a dipolar relaxation process necessitate some modification of this view. The difference in entropy of activation as well as the slightly higher energy of activation for the dielectric process suggest a possible mechanism. It seems reasonable that motions of the hydrocarbon segments might be accompanied by motions of the polar amide groups. Because of intermolecular hydrogen bonding, however, such combined motions would be less likely and would also involve a somewhat higher activation energy to break hydrogen bonds. A different entropy for the activated complex would be expected in the two cases. Thus, while the mechanical measurements undoubtedly “see” the relaxation of polar groups to some extent, the much more active motions of the hydrocarbon segments overshadow the process observed in the dielectric measurements.

The hypothesis that this relaxation process is due to motions of molecular end groups can be almost eliminated on the basis of two arguments. First, if such a mechanism were involved, the dielectric and mechanical relaxation processes should coincide at any given temperature and frequency, which is not the case. Second, the magnitude of the polarization associated with this process is not inversely proportional to molecular weight, at least within the limited range covered in the nylon 610 data presented here. However, it is difficult to separate effects of crystallinity and molecular weight. Therefore, the latter argument is not proven, and further work is in progress with other polyamide specimens covering a wider range of molecular weights. It should be pointed out that it is not a simple matter to relate the magnitude of the polarization with the known type and number of polar end groups since in this hydrogen bonded material, the relationship between dipole moment and polarizability cannot be predicted with sufficient accuracy.

## 4. Conclusions

Four relaxation phenomena have been identified in 610 and 66 nylon: (1) Above about 80 °C a dipolar relaxation process is apparent in both polymers. The earlier suggestion that this process does not occur in highly ordered regions is confirmed by comparison of dielectric, specific volume, and X-ray diffraction data. (2) There is a very-low-frequency polarization characterized by an extremely high loss index and dielectric constant above about 60 °C in both polyamides. This process, also observed by previous authors, is undoubtedly due to motions of amide group hydrogens involved in intermolecular hydrogen bonds. This process has been found to give rise to electret formation in these polyamides. (3) There is a relaxation process observed at room temperature at about 10 kc/s. This is evidently a result of the presence of water and, in one case, was almost completely absent after prolonged drying. This process has been shown to be the dielectric manifestation of the “*β* process” observed in dynamic mechanical measurements. (4) There is a low temperature relaxation process closely related to the “*γ* process” observed in mechanical measurements. This process, observed in both polyamides, necessarily involves dipolar motions and probably requires modification of the proposal that the “*γ* process” involves only motions of the hydrocarbon segments in these polymers.

## Figures and Tables

**Table 1:** Sample descriptions and specific volumes

Number	Mode of preparation and thermal history	Specific volume at 23 °C	% Crystallinity (see text)	Thickness	Area

		*cm*^3^/*g*		*cm*	*cm*^2^
66–1	Melted in vacuum, compression molded, heated in vacuum for three days at 130 °C.	0.8709	56	0.3540	10.372
66–2	Same as 66–1.	.8637	62	.3823	5.0623
66–3	Same as 66–1.	.8676	58	.1461	10.525
66–4	Cut from same disc as 66–3	.8676	58	.1485	10.535
66–6	Melted in vacuum, compression molded. (Heated in vacuum for subsequent measurements, no perceptible change in *V_8_*.)	.8685	58	.3365	7.9118
610–1	Injection molded, stored one month over P_2_O_5_.	.9289	34	.3197	7.285
	After heating in vacuum to 110 °C for three days.	.9287	34	——	——
	After heating to 210 °C for one day.	.9138	50	——	——
610–2	Melted in vacuum, heated five days in vacuum at 130 °C.	.9181	46	.2545	7.905
610–7	Injection molded, stored over P_2_O_5_.	.9289	34	——	——
	After drying at 130 °C in vacuum for five days.	.9248	38	——	——
	After heating in vacuum at 210 °C for one day.	.9156	48	.2924	7.785
610–8	Melted in vacuum, heated five days at 130 °C in vacuum.	.9150	49	.1745	10.616
610–9	Injection molded and heated three days at 110 °C.	——	——	.1578	11.445

**Table 2:** Intrinsic viscosities In *m-cresol at* 30 °C.

Sample description	
66 Nylon samples:	
Chips, as received	1.18
Chips, dried in vacuum at 130 °C for 3 days	1.26
Vacuum melted, dried in vacuum at 130 °C for 3 days	1.69
Vacuum melted, dried in vacuum at 130 °C for 3 days	1.73
610 Nylon samples:	
Injection molded, as received	0.96
Injection molded, dried at 130 °C for 3 days	.96
Melted in vacuum, dried at 130 °C for 3 days	1.26
Melted in vacuum, dried at 210 °C for 1 day	1.33

**Table 3:** Dielectric constants and loss indexes of 66 nylon samples

	66–1											
	23 °C		51.3 °C		75.2 °C		99 °C		125.4 °C		149.6 °C	
	
*Frequency*	*ϵ*′	*ϵ*″×*10*^4^	*ϵ*′	*ϵ*″×*10*^4^	*ϵ*′	*ϵ*″	*ϵ*′	*ϵ*″	*ϵ*′	*ϵ*″	*ϵ*′	*ϵ*″
50 c/s	3.570	278.7	3.926	842	7.526	1.969						
100	3.560	327.4	3.900	771	6.961	1.529	20.56	19.04				
200	3.546	346.2	3.873	685	6.487	1.101	16.94	11.77	50.72	100.1		
500	3.522	397.6	3.834	666	5.991	0.8045	13.27	6.49				
1 kc/s	3.505	421	3.800	683	5.728	.6765	11.85	4.30	26.78	29.38	60.32	140.1
2	3.486	458	3.780	714	5.495	.584	10.91	2.96	21.78	18.60	43.62	82.0
5	3.455	499	3.733	795	5.186	.499	9.82	1.95	16.44	9.78	30.55	39.7
10	3.432	514	3.698	841	4.987	.455	9.24	1.60	14.55	6.118	24.82	24.3
20	3.411	516	3.661	865	4.810	.428	8.717	1.47	13.44	4.035	19.31	14.92
50	3.376	541	3.664	919	4.550	.393	7.881	1.34	12.22	2.445	15.89	7.66
100	3.352	566	3.563	948	4.389	.374	7.315	1.34	11.63	1.976	14.51	4.79
310	3.292	570	3.493	902	4.144	.2959	6.516	1.102	10.75	1.496		
1 Mc/s	3.261	612	3.436	901	3.963	.2560	5.558	0.953	9.43	1.603		
3.2	3.211	647	3.374	888	3.743	.2200	4.817	.750	7.79	1.678		
10	3.165	708	3.305	921	3.593	.2041	4.313	.593	6.19	1.505		

	66–2									
	23.5 °C		51.3 °C		61.4 °C		66.0 °C		71.3 °C	
	
*Frequency*	*ϵ*′	*ϵ″×*10**^4^	*ϵ*′	*ϵ″×*10**^4^	*ϵ*′	*ϵ″×10*^4^	*ϵ*′	*ϵ″×10*^4^	*ϵ*′	*ϵ″×10*^4^
50 c/s	3.641	328	3.956	840	4.538	3120	5.067	5600	5.770	9330
100	3.627	365	3.931	763	4.441	2590	4.892	4560	5.491	7290
200	3.612	372	3.910	716	4.357	2040	4.754	3540	5.265	5570
500	3.587	401	3.874	699	4.249	1670	4.575	2760	4.979	4170
1 kc/s	3.572	438	3.845	709	4.187	1500	4.474	2400	4.826	3510
2	3.549	451	3.817	755	4.130	1430	4.388	2160	4.700	3120
5	3.516	480	3.770	801	4.044	1390	4.262	1990	4.524	2750
10	3.494	496	3.735	827	3.985	1380	4.181	1890	4.416	2580
20	3.473	476	3.700	846	3.928	1350	4.106	1860	4.316	2460
50	3.441	499	3.642	859	3.838	1330	3.985	1790	4.162	2320
100	3.418	529	3.608	885	3.776	1340	3.905	1770	4.065	2260
310	3.293	526	3.481	851	3.635	1240	3.693	1620	3.868	1980
1 Mc/s	3.276	534	3.455	850	3.559	1180	3.625	1440	3.749	1820
3.2	3.259	584	3.404	858	3.491	1120	3.574	1330	3.605	1610
10	3.191	683	3.303	962	3.389	1170	3.438	1300	3.503	1540

	66–2 (Cont.)											
	75.8 °C		80.0 °C		99.8 °C		125.9 °C		150.2 °C		175.2 °C	
	
*Frequency*	*ϵ*′	*ϵ*″	*ϵ*′	*ϵ*″	*ϵ*′	*ϵ*″	*ϵ*′	*ϵ*″	*ϵ*′	*ϵ*″	*ϵ*′	*ϵ*″
50 c/s	6. 989	1.677	8.497	2.798	22.99	23.76						
100	6.508	1.253	7.698	2.052	18.60	15.29	40.64	168.3				
200	6.137	0.9413	7.122	1.456	15.24	9.962	36.44	89.13				
500	5.694	.6750	6.468	0.997	12.284	5.920	28.73	44.42	44.68	262.62		
1 kc/s	5.461	.5642	6.135	.803	10.828	3.902	23.70	26.40	37.67	138.23		
2	5.270	.4963	5.862	.690	9.857	2.659	19.297	16.61	32.57	74.09	41.35	322.31
5	4.992	.4257	5.477	.577	8.992	1.767	15.199	9.157	26.21	35.81	35.05	135.22
10	4.827	.3862	5.243	.5228	8.461	1.421	13.288	5.692	21.447	21.63	30.86	73.47
20	4.682	.3645	5.042	.4847	7.969	1.250	12.061	3.667	17.647	13.210	26.15	40.71
50	4.459	.3361	4.745	.4399	7.150	1.204	11.082	2.223	14.461	7.053	17.98	18.87
100	4.320	.3193	4.562	.4120	6.535	1.009	10.510	1.756	12.804	4.334	16.80	12.55
310	4.087	.2735	4.245	.3526	6.13	1.030	9.80	1.513	12.15	2.141	13.90	5.295
1 Mc/s	3.909	.2411	4.008	.2968	5.37	0.868	8.63	1.623	11.23	1.572	12.63	2.320
3.2	3.663	.2073	3.779	.2467	4.70	.679	7.41	1.656	10.24	1.704	11.94	1.501
10	3.570	.1839	3.601	.2181	4.18	.522	5.97	1.497	8.87	2.091	11.07	1.726

	66–3													
	23 °C		0.3 °C		−25.1 °C		−49.5 °C		−79.6 °C		−98.0 °C		23.2 °C	
	
*Frequency*	*ϵ*	*ϵ*″×10^4^	*ϵ*′	*ϵ*″×10^4^	*ϵ*′	*ϵ*″×10^4^	*ϵ*′	*ϵ*″×10^4^	*ϵ*′	*ϵ*″×10^4^	*ϵ*′	*ϵ*″×10^4^	*ϵ*′	*ϵ*″×10^4^
50 c/s	3.554	213	3.508	339	3.421	353	3.337	323	3.235	287	3.162	282	3.535	218
100	3.545	239	3.495	355	3.405	352	3.323	317	3.223	293	3.149	285	3.526	251
200	3.534	275	3.480	380	3.390	357	3.310	328	3.210	311	3.137	285	3.515	291
500	3.515	326	3.456	394	3.367	362	3.289	336	3.190	324	3.118	280	3.496	347
1 kc/s	3.500	360	3.439	408	3.351	363	3.275	348	3.176	335	3.106	266	3.480	375
2	3.484	386	3.422	412	3.336	373	3.260	357	3.162	341	3.095	260	3.463	400
5	3.458	422	3.380	427	3.312	382	3.237	382	3.139	341	3.078	235	3.435	441
10	3.439	435	3.378	437	3.295	397	3.221	396	3.124	330	3.068	211	3.416	453
20	3.420	444	3.360	442	3.278	413	3.204	406	3.110	309	3.059	197	3.396	457
50	3.391	469	3.331	461	3.251	440	3.176	418	3.090	292	3.047	176	3.366	473
100	3.371	484	3.311	481	3.232	461	3.157	415	3.078	269	3.040	158	3.346	489
310	3.364	488	3.302	501	3.227	465	3.138	366	3.062	223	3.048	129	3.324	488
1 Mc/s	3.308	535	3.248	534	3.172	457	3.108	331	3.039	189	3.026	104	3.268	529
3.2	3.265	583	3.205	556	3.136	421	3.078	285	3.032	156	3.013	87	3.238	570
10	3.215	644	3.163	542	3.112	379	3.069	248	3.021	126	3.004	78	3.196	625

	66–4		66–3						66–2			
	
	23 °C		23 °C		23 °C with .72% H_2_O		22.8 °C redried		23 °C		100.6 °C with .69% H_2_O	
	
*Frequency*	*ϵ*′	*ϵ*″×10^4^	*ϵ*′	*ϵ*″×10^4^	*ϵ*′	*ϵ*″×10^4^	*ϵ*′	*ϵ*″×10^4^	*ϵ*′	*ϵ*″×10^4^	*ϵ*′	*ϵ*″
50 c/s	3.554	213	3.542	214	4.088	1348	3.533	204	3.753	624	……	……
100	3.545	239	3.533	257	4.036	1248	3.525	232	3.723	620	……	……
200	3.534	275	3.523	278	3.983	1219	3.516	272	3.697	667	……	……
500	3.515	326	3.504	330	3.913	1247	3.497	321	3.652	686	……	……
1 kc/s	3.500	360	3.489	365	3.856	1265	3.482	348	3.620	706	14.15	7.539
2	3.484	386	3.473	394	3.799	1289	3.467	373	3.589	745	12.26	5.041
5	3.458	422	3.445	431	3.721	1313	3.442	407	3.538	794	10.66	3.027
10	3.439	435	3.426	445	3.661	1316	3.424	417	3.503	804	9.827	2.182
20	3.420	444	3.407	447	3.602	1288	3.406	429	3.467	796	9.188	1.676
50	3.391	469	3.379	464	3.526	1212	3.379	449	3.412	803	8.478	1.410
100	3.371	479	3.359	472	3.471	1145	3.360	468	3.376	763	7.904	1.343
310	3.364	488	……	……	3.418	939	3.324	471	3.29	687	7.18	1.282
1 Mc/s	3.308	535	……	……	3.337	805	3.273	514	3.25	611	6.21	1.193
3.2	3.265	583	……	……	3.276	715	3.232	589	3.20	573	5.26	0.988
10	3.215	584	……	……	3.229	628	3.180	719	3.12	569	4.49	.695

	66–6													
	23 °C not dried		100.5 °C not dried		23 °C vacuum dried		100 °C vacuum dried		111.0 °C vacuum dried		120.3 °C vacuum dried		130.3 °C vacuum dried	
	
*Frequency*	*ϵ*′	*ϵ*″×*10*^4^	*ϵ*′	*ϵ*″	*ϵ*′	*ϵ*″×*10*^4^	*ϵ*′	*ϵ*″	*ϵ*′	*ϵ*″	*ϵ*′	*ϵ*″	*ϵ*′	*ϵ*″
50 c/s	3.650	406	……	……	3.600	297	……	……	……	……	……	……	……	……
100	3.628	440	23.779	22.08	3.585	322	19.66	15.95	……	……	……	…….	…….	……
200	3.608	499	18.924	14.35	3.571	372	16.02	10.46	……	…….	……	……	……	…….
500	3.573	564	14.956	8.273	3.547	407	13.02	6.01	……	……	…….	……	…….	…….
1 kc/s	3.547	610	13.055	5.598	3.529	430	11.58	3.98	15.69	9.19	20.93	18.35	28.96	36.48
2	3.518	668	11.758	3.817	3.510	465	10.52	2.77	14.11	5.80	17.23	11.66	22.89	22.39
5	3.472	741	10.528	2.410	3.478	495	9. 649	1.89	11.96	3.476	14.314	6.587	18.151	12.22
10	3.435	752	9.899	1.891	3.457	510	9.057	1.55	11.12	2.417	12.940	4.090	15.170	7.429
20	3.402	743	9.263	1.617	3.434	529	8.503	1.38	10.45	1.887	12.020	2.814	13.710	4.709
50	3.355	731	8.471	1.477	3.400	548	7.784	1.28	9.671	1.579	11.153	1.950	12.537	2.805
100	3.322	704	7.850	1.457	3.376	571	7.221	1.27	9.060	1.547	10.558	1.748	11.883	2.133
310	3.261	584	6.992	1.329	3.381	566	6.463	1.14	8.138	1.499	9.720	1.624	11.117	1.663
1 Mc/s	……	……	5.916	1.151	3.280	621	5.573	0.985	6.917	1.443	8.373	1.728	9.920	1.880
3.2	3.156	737	5.102	.891	3.237	658	4.869	.770	5.82	1.216	6.970	1.643	8.377	1.931
10	3.098	712	4.455	.691	3.160	665	4.326	.566	4.91	0.893	5.633	1.282	6.68	1.737

**Table 4:** Dielectric constants and loss indexes of 610 nylon samples

	610–1						After vacuum drying five days at 130 °C				After 1 day vacuum drying at 210 °C	

	23 °C		80.7 °C		100 °C		23 °C		80.1 °C		80.2 °C	

	*ϵ*′	*ϵ*″×*10*^4^	*ϵ*′	*ϵ*″	*ϵ*′	*ϵ*″	*ϵ*′	*ϵ*″×*10*^4^	*ϵ*′	*ϵ*″	*ϵ*′	*ϵ*″
	
*Frequency*												
50 c/s	3.665	561	16.34	6.780	……	……	3.558	526	……	…….	……	……
100	3.640	627	15.05	4.480	……	……	3.533	574	……	……	……	……
200	3.614	683	13.89	3.150	……	…….	3.509	601	……	…….	……	……
500	3.569	748	12.843	2.180	……	……	3.468	654	……	…….	…….	……
1 kc/s	3.535	782	12.165	1.780	15.990	4.570	3.438	660	9.786	1.230	8.311	0.950
2	3.501	829	11.543	1.600	14.954	3.070	3.410	687	9.255	1.250	7.885	.970
5	3.448	888	10.749	1.530	14.046	2.030	3.365	685	8.546	1.300	7.307	1.020
10	3.407	895	10.078	1.560	13.498	1.690	3.336	674	7.940	1.331	6.825	1.040
20	3.367	894	9.374	1.610	12.865	1.560	3.306	645	7.321	1.332	6.338	1.050
50	3.308	879	8.392	1.680	12.059	1.640	3.265	641	6.518	1.290	5.702	1.010
100	3.270	844	7.583	1.720	11.330	1.840	3.236	645	5.931	1.240	5.219	0.960
310	3.138	677	6.480	1.490	10.064	2.090	3.163	621	5.221	0.950	4.725	.752
1 Mc/s	3.109	630	5.337	1.170	8.137	2.200	3.104	625	4.563	.730	4.169	.570
3.2	3.094	617	4.562	0.840	6.407	1.840	3.064	650	4.081	.510	3.776	.410
10	3.009	629	4.036	.561	5.142	1.285	3.024	671	3.697	.355	3.510	.284

610–2														
	23 °C		80.9 °C		+0.2 °C		−25.3 °C		−50.0 °C		−83.1 °C		−106.2 °C	

	*ϵ*′	*ϵ″×10*^4^	*ϵ*′	*ϵ″*	*ϵ*′	*ϵ″×10*^4^	*ϵ*′	*ϵ″×10*^4^	*ϵ*′	*ϵ″×10*^4^	*ϵ*′	*ϵ″×10*^4^	*ϵ*′	*ϵ″×10*^4^
	
*Frequency*														
50 c/s	3.324	393	……	……	3.311	380	3.212	364	3.119	312	3.031	283	2.943	245
100	3.306	429	……	……	3.293	401	3.195	357	3.107	314	3.020	293	2.935	244
200	3.287	450	……	……	3.276	419	3.180	363	3.096	324	3.009	289	2.922	240
500	3.257	482	……	……	3.247	406	3.157	341	3.079	317	2.991	301	2.913	228
1 kc/s	3.236	495	9.177	1.153	3.229	403	3.143	329	3.067	317	2.978	320	2.903	226
2	3.214	504	8.689	1.149	3.211	400	3.128	326	3.055	317	2.965	316	2.896	206
5	3.180	524	8.035	1.170	3.185	399	3.108	329	3.037	354	2.951	325	2.884	199
10	3.157	514	7.499	1.200	3.169	399	3.093	328	3.024	361	2.934	312	2.877	185
20	3.136	495	6.942	1.200	3.152	390	3.080	328	3.010	374	2.923	285	2.870	161
50	3.102	495	6.207	1.160	3.127	391	3.058	350	2. 989	397	2.907	266	2.861	149
100	3.082	496	5.684	1.120	3.111	402	3.043	377	2.972	405	2.893	247	2.856	140
310	3.040	469	5.060	0.870	3.063	413	2.987	382	2.932	351	2.881	181	2.804	110
1 Mc/s	2.978	499	4.420	.667	2.997	446	2.958	413	2.847	318	2.833	145	2.789	76
3.2	2.939	520	4.010	.480	2.983	472	2.954	395	2.925	280	2.833	127	2.785	72
10	2.898	539	3.654	.341	2.949	470	2.880	359	2.832	238	2.807	124	2.763	73

610–7				
After vacuum drying at 210 °C for one day				
	23 °C		79.5 °C	

	*ϵ*′	*ϵ*″×*10*^4^	*ϵ*′	*ϵ*″
	
*Frequency*				
50 c/s	3.381	399	10.164	1.466
100	3.363	430	9.727	1.210
200	3.344	464	9.288	1.061
500	3.313	478	8.791	0.990
1 kc/s	3.291	488	8.391	.984
2	3.271	505	7.938	.998
5	……	504	7.367	1.055
10	3.215	497	6.899	1.072
20	3.194	483	6.376	1.074
50	3.163	486	5.738	1.040
100	3.141	478	5.284	0.973
310	3.067	465	4.721	.766
1 Mc/s	3.008	479	4.212	.578
3.2	2.991	510	3.770	.403
10	2.953	581	2.981	.286

610–8													
	23 °C		0 °C		−26.1 °C		−50.1 °C		−75.6 °C		−100.1 °C		80.5 °C
	
*Frequency*	*ϵ*′	*ϵ*″×*10*^4^	*ϵ*′	*ϵ*″×*10*^4^	*ϵ*′	*ϵ*″×*10*^4^	*ϵ*′	*ϵ*″×*10*^4^	*ϵ*′	*ϵ*″×*10*^4^	*ϵ*′	*ϵ*″×*10*^4^	*ϵ*″
50 c/s	3.420	455	3.254	364	3.189	350	3.120	304	3.048	241	2.972	238	……
100	3.409	487	3.241	378	3.173	348	3.107	287	3.038	249	2.962	238	……
200	3.388	504	3.227	394	3.159	346	3.093	280	3.028	252	2.953	238	……
500	3.355	536	3.205	399	3.136	341	3.076	281	3.011	272	2.938	227	……
1 kc/s	3.331	543	3.188	395	3.123	323	3.062	283	2.999	282	2.927	222	1.178
2	3.308	546	3.174	390	3.109	317	3.050	286	2.988	285	2.918	221	1.184
5	3.273	559	3.150	398	3.089	324	3.032	312	2.968	304	2.904	209	1.208
10	3.248	549	3.132	392	3.075	320	3.018	319	2.954	297	2.896	188	1.238
20	3.224	529	3.116	381	3.062	320	3.004	329	2.941	285	2.888	169	1.242
50	3.192	535	3.092	393	3.041	349	2.982	356	2.923	276	2.878	155	1.203
100	3.169	535	3.076	393	3.026	365	2.965	359	2.911	257	2.871	139	1.130
310	3.155	503	3.035	393	2.981	374	2.923	332	2.871	221	2.847	119	……
1 Mc/s	3.054	509	2.987	432	2.941	398	2.840	313	2.824	195	2.815	97	……
3.2	3.030	523	2.963	474	2.901	391	2.859	286	2.832	171	2.812	85	……
10	2.986	502	2.937	494	2.886	372	2.837	240	2.821	135	2.821	120	……

610–9										
	23° C		0 °C		−25.3 °C		−50.9 °C		−75.2 °C	
	
*Frequency*	*ϵ*′	*ϵ*″×*10*^4^	*ϵ*′	*ϵ*″×*10*^4^	*ϵ*′	*ϵ*″×*10*^4^	*ϵ*′	*ϵ*″×*10*^4^	*ϵ*′	*ϵ*″×*10*^4^
50 c/s	3.507	454	3.345	462	3.229	446	3.133	350	3.050	272
100	3.488	498	3.324	485	3.209	442	3.119	338	3.037	279
200	3.466	537	3.304	493	3.191	425	3.105	329	3.025	287
500	3.429	584	3.271	515	3.165	405	3.085	325	3.006	307
1 kc/s	3.402	620	3.249	509	3.147	385	3.071	326	2.993	315
2	3.376	626	3.226	499	3.131	376	3.057	328	2.979	324
5	3.322	641	3.195	496	3.108	376	3.036	355	2.957	342
10	3.294	644	3.173	475	3.092	372	3.020	370	2.942	338
20	3.267	624	3.154	458	3.077	374	3.004	379	2.927	320
50	3.227	616	3.125	460	3.052	404	2.978	408	2.905	309
100	3.200	610	3.105	461	3.035	428	2.960	411	2.892	275
310	3.130	565	3.054	448	2.969	431	2.904	370	2.846	239
1 Mc/s	3.071	577	2.996	502	2.924	457	2.865	342	2.811	206
3.2	3.029	608	2.957	533	2.885	433	2.832	297	2.791	175
10	2.991	637	2.921	540	2.851	383	2.821	253	2.797	149

## Footnotes

1 Figures in brackets indicate the literature references at the end of this paper.

2 This is so because electrode polarization would introduce a polarization confined to the immediate area of the electrodes, independent of sample thickness. If this effect were pronounced, it would outweigh the effect of bulk polarization. Thus the measured dielectric constant would equal some capacitance caused by electrode polarization divided by the equivalent vacuum capacitance of the volume occupied by the sample: ϵ′ = *C_p_*/*C_v_* The capacitance, due to electrode polarization, *C_p_*, would be independent of sample thickness; the equivalent vacuum capacitance, *C_v_*, is inversely proportional to sample thickness. Therefore, if pronounced electrode polarization occurs, the measured dielectric constant should tend to be proportional to thickness.
